# Urban evolution comes into its own: Emerging themes and future directions of a burgeoning field

**DOI:** 10.1111/eva.13165

**Published:** 2020-12-07

**Authors:** Lindsay S. Miles, Elizabeth J. Carlen, Kristin M. Winchell, Marc T. J. Johnson

**Affiliations:** ^1^ Department of Biology University of Toronto Mississauga Mississauga ON Canada; ^2^ Centre for Urban Environments University of Toronto Mississauga Mississauga ON Canada; ^3^ Department of Biology Fordham University Bronx NY USA; ^4^ Department of Biology Washington University St. Louis MO USA

**Keywords:** Anthropocene, cities, eco‐evolutionary dynamics, gene flow, urban adaptation

## Abstract

Urbanization has recently emerged as an exciting new direction for evolutionary research founded on our growing understanding of rapid evolution paired with the expansion of novel urban habitats. Urbanization can influence adaptive and nonadaptive evolution in urban‐dwelling species, but generalized patterns and the predictability of urban evolutionary responses within populations remain unclear. This editorial introduces the special feature “Evolution in Urban Environments” and addresses four major emerging themes, which include: (a) adaptive evolution and phenotypic plasticity via physiological responses to urban climate, (b) adaptive evolution via phenotype–environment relationships in urban habitats, (c) population connectivity and genetic drift in urban landscapes, and (d) human–wildlife interactions in urban spaces. Here, we present the 16 articles (12 empirical, 3 review, 1 capstone) within this issue and how they represent each of these four emerging themes in urban evolutionary biology. Finally, we discuss how these articles address previous questions and have now raised new ones, highlighting important new directions for the field.

## 1 INTRODUCTION

It has recently been established that urbanization can influence adaptive and nonadaptive evolution in urban organisms. This special issue presents the latest research, identifies existing gaps, and provides a vision for future urban evolutionary research. The overwhelming conclusion presented in this issue is that urban evolutionary change is becoming an increasingly prominent and important process shaping the contemporary evolution of species throughout the world. This bold assertion stems from three related phenomena. First, the global human population continues to increase and concentrate in urban areas (United Nations, 2018), with 68% of the world's population (10 billion people) predicted to live in urban areas by 2050. Second, although the global extent of urbanization remains relatively concentrated, the growth of urban areas continues rapidly throughout the world such that the majority of terrestrial ecosystems are impacted in some way by human activity (Lewis & Maslin, 2015; Liu et al., 2020; Seto et al., 2012). Finally, urban development creates novel environments over compressed temporal and spatial scales, creating the conditions to drive rapid adaptive and nonadaptive evolutionary change (Donihue & Lambert, 2014; Johnson & Munshi‐South, 2017; Szulkin et al., 2020).

This editorial provides an introduction to the special feature “Evolution in Urban Environments.” We first describe urbanization in the context of biological studies and give a brief history of research on urban evolutionary biology. We then provide an overview of current themes within urban evolution in light of the research presented in this issue. We end with a look to emerging themes and challenges for future research.

### 1.1 What is urbanization?

For the purpose of this special issue, we operationally define urban areas as dense human populations, typified by cities and the infrastructure associated with these areas (e.g., buildings, roads, and landscape changes). Beyond this simple definition, the process of urban development (i.e., urbanization) is inherently multidimensional and, in some respects, difficult to define. Yet, there are changes to the natural environment that researchers generally agree characterize urban ecosystems. Urban environments tend to have increased impervious surfaces, higher human population density, elevated temperatures (i.e., the urban heat island effect), higher pollution levels, and highly fragmented habitats. These dramatic changes to the natural landscape present novel challenges for organisms. Consequently, urbanization can affect eco‐evolutionary dynamics, including adaptive and nonadaptive evolution of urban populations, as well as feedbacks onto ecosystems.

**Figure 1:**
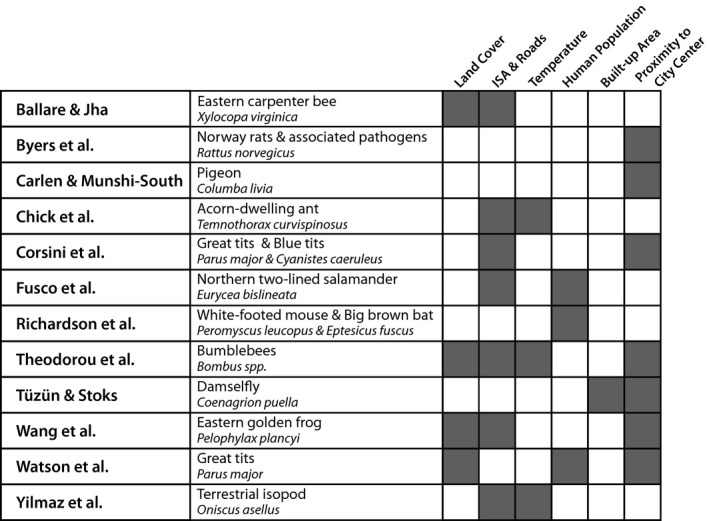
Researchers use a wide variety of metrics to describe urban habitats, and different metrics are appropriate for different species and to address different questions. The 12 empirical papers in this special issue used the following metrics to describe urbanization and to analyze phenotypic and genotypic variation: land cover and use, impervious surface cover (ISA) and roads, temperature, human population density, built‐up land cover, and proximity to the city center or within metropolitan boundaries (gray boxes indicate the metric(s) used in each study)

Urbanization is not a uniform process, and urban areas (hereafter called “cities” for simplicity) can vary substantially from one another. The following are a few examples of the local characteristics that vary within and between cities: the age of a city, the extent and pattern of development, policies on urban planning, control of urban wildlife, regional societal practices, and historical and contemporary socioeconomic patterns (including structural racism). This topic has received substantial attention recently, and we recommend Szulkin et al., 2020 (chapter 2), Schell, Dyson, et al. (2020), and the UN's 2018 report on global urbanization for more in‐depth discussion of how to define and measure urbanization. The specific aspects of urban environmental change that are most relevant to the evolution of a particular species are likely to vary and should be chosen carefully to reflect the specific characteristics of a city and biology of a focal organism, as is reflected by the diverse metrics used to characterize urban environments in this special issue (Figure 1).

### 1.2 Urban evolution: current state of knowledge

The nascent field of urban evolutionary biology has in recent years provided myriad examples of evolutionary changes associated with urbanization in a wide variety of taxa and at all levels of biological hierarchy (i.e., functional, phenotypic, regulatory, genomic). The exponential growth of this field has yielded important insights into fundamental ecological and evolutionary questions (Rivkin et al., 2019; Santangelo et al., 2018; Szulkin et al., 2020). As empirical examples of evolutionary change have accumulated, so too have the retrospective syntheses, providing important perspectives on key themes and future directions for the field.

Common themes from these syntheses include identifying phenotypically plastic versus heritable responses, evaluation of both adaptive and nonadaptive evolution, and assessing if urbanization has repeatable effects on the ecology and evolution of urban‐dwelling species (Alberti, 2015; Donihue & Lambert, 2014; Johnson & Munshi‐South, 2017; Lambert & Donihue, 2020; McDonnell & Hahs, 2015; Miles et al., 2019; Rivkin et al., 2019; Schell, 2018; Schmidt et al., 2020; Smith & Bernatchez, 2008; Szulkin et al., 2020; Thompson et al., 2018). Additionally, these syntheses indicate that when evaluating adaptive and nonadaptive evolution, heterogeneity across the landscape and other landscape features are important factors to consider (Lambert & Donihue, 2020; Miles et al., 2019; Rivkin et al., 2019; Schell, 2018; Schmidt et al., 2020; Szulkin et al., 2020). While each of these reviews, syntheses, and even special issues repeatedly highlight these common themes, there is not yet consensus of how urbanization is shaping evolution. Indeed, the field of urban evolutionary biology is still in its infancy, with the majority of the empirical research and theoretical research published in the last 10 years (Rivkin et al., 2019).

Due to the rapid growth of urban evolutionary biology, there are many questions that have yet to be addressed. The most pertinent of these outstanding questions ask: Are the evolutionary responses to urbanization predictable? Specifically, what is the prevalence of convergence at the genetic and phenotypic level across urban environments and among different species? If we are able to identify the drivers of evolution and predict responses to urbanization, then perhaps we will be better able to apply this information to conservation, land‐use management (Alberti, 2015; Johnson & Munshi‐South, 2017; Lambert & Donihue, 2020; Rivkin et al., 2019), and the intersection of human socioeconomic variables (e.g., systemic racism, poverty, health) and ecology and evolutionary change (Schell, Dyson, et al., 2020). This special issue, “Evolution in Urban Environments,” takes a step in this direction by addressing these questions.

## 2 THE SPECIAL ISSUE

The state of knowledge in the burgeoning field of urban evolutionary ecology is rapidly changing. Retrospective and prospective review papers provide valuable and continuing feedback to shape the field, but it is the results from empirical research conducted on urban‐dwelling organisms that are critical to answer questions, address gaps, and push the field in new directions. With this special issue, our goal is to synthesize the current state of the field, address existing gaps in knowledge, and inspire new directions of research and application.

**Figure 2:**
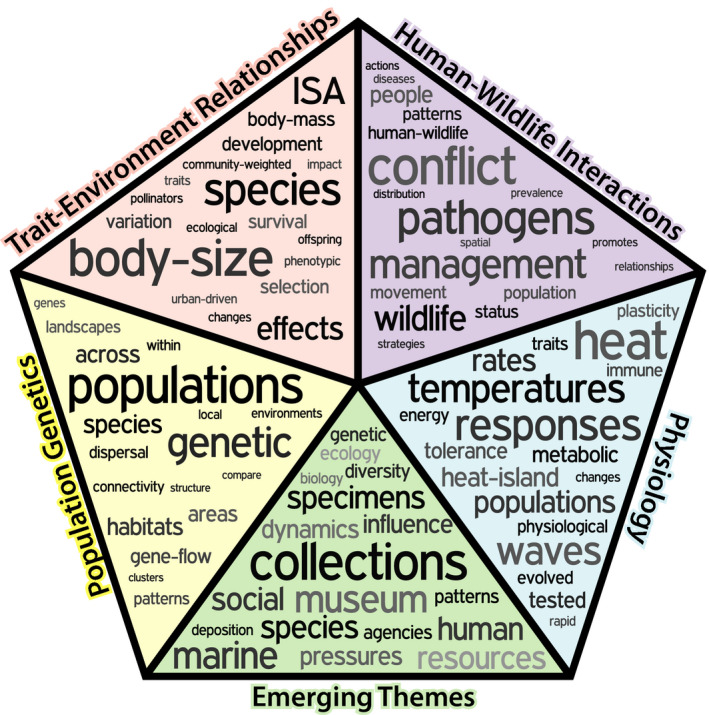
We identified four focal topics from the empirical contributions (population genetics—yellow, trait–environment relationships—pink, human–wildlife interactions—purple, physiology—blue) as well as emerging themes from the reviews and capstone paper (green). These five themes are presented here with the top 19 words from the abstracts, with word size relative to the prevalence across the abstracts in each group

**Figure 3:**
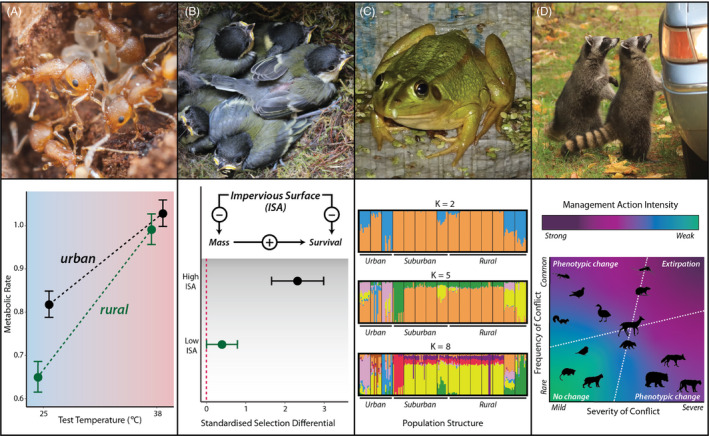
This special issue includes empirical work from four main themes related to evolution in response to urbanization. (a) Adaptive evolution of physiology: Chick et al. (2020) found acorn ants exhibit elevated metabolic rates, but this difference diminishes under acute thermal stress and is associated with increased resource acquisition. Top: Acorn Ants, *Temnothorax curvispinosus*, Lauren Nichols; bottom: modified figure 2A from Chick et al. (2020). (b) Adaptive evolution of trait–environment relationships: Corsini et al. (2020) examined relationships between impervious surface cover (ISA) and growth rate, body size, and survival, finding strong selection on body size in habitats with more impervious surface cover. Top: *Parus major*, Michela Corsini; bottom: modified figures 3 and 4 from Corsini et al. (2020). (c) Population Genetics: Wang (2020) found bottlenecks occurred in all populations prior to recent urbanization, but there was genetic structure along the urban to rural gradient. Top: *Pelophylax plancy*i, photo credit Wei Xu; bottom: modified figure 4 from Wang (2020). (d) Human–wildlife interactions: Schell, Stanton, et al. (2020) presents a perspective that integrates human–wildlife interaction, wildlife management and urban evolution to address how organisms adapt to urban environments while experiencing socio‐ecological processes. Top: Racoons, *Procyon lotor*, Zachary Hawn; bottom: modified figure 4 from Schell, Stanton, et al. (2020)

The special issue comprises 16 papers, including 12 empirical contributions, three reviews, and one capstone perspective. The empirical studies represent diverse taxonomic groups from urbanized habitats primarily in North America and Europe, with an additional study in China. These papers represent four major themes: (a) adaptive evolution via physiological responses to urban climate, (b) adaptive evolution via phenotype–environment relationships in urban habitats, (c) population connectivity and genetic drift, and (d) human–wildlife interactions (Figure 2). In addition, the reviews and capstone paper introduce emerging themes to help guide future research on topics as diverse as marine environments, use of natural history museum specimens, and the integration of socioeconomic processes into eco‐evolutionary dynamics (Figure 2). We provide a brief summary of these contributions in the sections below and conclude with an assessment of the progress the papers in this special issue make in addressing questions and gaps previously identified (Figure 3; Alberti, 2015; Donihue & Lambert, 2014; Johnson & Munshi‐South, 2017; Rivkin et al., 2019). We conclude with a forward‐looking assessment that identifies the directions the field is heading to address remaining gaps in knowledge.

### 2.1 Physiological responses to urban climate

Recent work has advanced our understanding of the evolutionary impacts of urban heat islands on animal physiology (Brans et al., 2018; Campbell‐Staton et al., 2020; Chick et al., 2019; Diamond et al., 2018). Yet, the more we know about physiological responses to the altered climate of urban environments, the more new questions seem to emerge. Three studies in this special issue push our understanding of urban physiological responses in new directions by examining adaptive responses to urban temperatures in physiological traits and by explicitly examining the relative contributions of evolved versus phenotypically plastic physiological changes using a common garden approach (Chick et al., 2020; Tüzün & Stoks, 2020; Yilmaz et al., 2020). Their findings highlight the complex and interrelated nature of traits affected by urban heat islands, with different evolutionary versus plastic mechanisms underlying different physiological responses.

Previous studies have established that urban organisms are able to tolerate elevated temperatures typical of urban environments and have found both phenotypically plastic and genetic underpinnings for this variation (Brans et al., 2018; Campbell‐Staton et al., 2020; Diamond et al., 2018). Yilmaz et al. (2020) add to this growing body of evidence for thermal adaptation by exploring phenotypic plasticity and adaptive evolution of both heat and cold tolerance, as well as desiccation tolerance, in the terrestrial isopod *Oniscus asellus*. Urban isopods exhibited evolved differences in increased heat tolerance compared to their rural counterparts, but not in cold or desiccation tolerance. Unlike many urban organisms, urban isopods exhibited no phenotypic plasticity in heat tolerance, but did exhibit a phenotypically plastic response of diminished cold tolerance. Although no directional shifts in body size or desiccation tolerance were observed between urban and rural populations in either “cool” or “hot” rearing conditions, larger individuals exhibited improved desiccation tolerance.

Little is known about consequences of elevated urban temperatures beyond thermal tolerance. Tüzün and Stoks (2020) focused on how relatively understudied traits related to fitness in the damselfly *Coenagrion puella* respond to heat waves. As hypothesized, both urban and rural populations experienced decreased survival and growth, decreased bioenergetic responses, and increased immune responsiveness under simulated heat waves. Urban populations also exhibited divergent patterns of decreased heat wave‐induced energy depletion compared to rural populations, suggesting an adaptive shift to tolerate the stronger and more frequent heat waves of urban environments. Overall, this paper highlights the importance of linking fitness‐related traits to thermal physiological adaptations.

Lastly, Chick et al. (2020) address another understudied aspect of the physiological effects of the urban climate: metabolic rate and resource acquisition in acorn ants (*Temnothorax curvispinosus*). Contrary to expectations, urban ants displayed elevated metabolic rates, a potentially maladaptive response under urban conditions necessitating increased energy input. This increase in energy consumption under warmer conditions could potentially lead to “metabolic meltdown” when ectotherms are unable to acquire sufficient food resources to meet elevated energetic demands (Huey & Kingsolver, 2019). However, the increase in metabolic rate diminished under acute heat stress resulting in similar metabolic rates in urban and rural ants. Moreover, urban ants exhibited faster resource acquisition across temperatures, an evolved response to elevated energetic demands. These findings suggest that potentially maladaptive physiological responses to urbanization may arise in response to the urban heat island effect, perhaps if selection is acting on correlated traits (e.g., heat tolerance). Alternatively, elevated metabolic rates may confer adaptive benefits in unanticipated ways.

### 2.2 Phenotype–environment relationships

Environmental change associated with urbanization can be a potent driver of natural selection and the evolution of urban‐adapted phenotypes. Studying the associations between landscape features (e.g., impervious surface, fragmentation, urban versus rural habitats) and genomic variation, phenotypes and/or fitness of organisms, can provide insight into the evolutionary processes (e.g., natural selection) and responses (e.g., adaptive evolution) caused by urban environmental change (Santangelo et al., 2020). This special feature provides several clear and powerful examples of how urban environments influence such evolutionary processes and patterns.

Bumblebees have become model organisms for studies of behavior, ecology, and evolution (Woodard et al., 2015). Habitat fragmentation has had a negative effect on the performance and survival of many native bees via the loss of foraging and nesting habitats (Carvell et al., 2017; Goulson et al., 2008). However, bumblebees are expected to show an increase in body size with increasing fragmentation that potentially increases survival (Gérard et al., 2018; Merckx et al., 2018). To test whether fragmentation caused by urbanization is associated with increased body size in bumblebees, Theodorou et al. (2020) sampled two short‐distance dispersing bumblebee species, *B. lapidarius* and *B. pascuorum*, and one long‐distance disperser, *B. terrestris*. Only the long‐distance disperser, *B. terrestris,* exhibited increased body size in urban areas across the study region. At smaller spatial scales, the two short‐distance dispersers, *B. lapidarius* and *B. pascuorum*, increased in body size with increased road density. Therefore, the increase in body size is scale‐dependent, with possible indirect positive effects on pollination ecosystem services. This study highlights the importance of spatial scale for different species and the cascading eco‐evolutionary feedbacks onto ecosystem function.

Although urbanization can have a positive influence on phenotypes linked to increased survival, this trend is not always the case. Unlike Theodorou et al. (2020), Corsini et al. (2020) found that urbanization reduces body size in two passerine birds. Specifically, growth rates of great tits (*Parus major*) and blue tits (*Cyanistes caeruleus*) were slower and offspring survival was lower in areas with increased impervious surface area. The mass at day 2 posthatching and the survival of fledglings were strongly positively associated in both species, which indicates that there is strong positive directional selection for increased body mass of young birds in urbanized environments. This study demonstrates the negative impact urbanization can have on development and survival of birds nesting in areas with high impervious surface. Although urbanization negatively influences the survival of great tits, there appears to be the potential for adaptive evolution in fledgling traits. Specifically, Watson (2020) identified putatively adaptive epigenetic markers in blood and liver tissue of fledgling great tits. DNA methylation sites in the liver were enriched within regulatory regions, suggesting that there is gene expression variation in metabolic processes between urban and forest birds that may increase fitness.

### 2.3 Population genetic patterns associated with urbanization

Gene flow and genetic drift may be influenced by urbanization either through “urban fragmentation” or “urban facilitation,” which encapsulate two hypotheses concerning the influence of urbanization on nonadaptive evolutionary processes and consequently the genetic structure of populations (Miles et al., 2019; Schmidt et al., 2020). Populations experiencing urban fragmentation are expected to exhibit greater genetic drift and reduced gene flow, leading to reduced genetic diversity within populations and increased genetic differentiation between populations, potentially leading to population declines (Miles et al., 2019; Schmidt et al., 2020). By contrast, populations experiencing urban facilitation are predicted to experience reduced genetic drift and greater gene flow within and between urban environments, potentially promoting increased genetic diversity within populations and reduced genetic differentiation between populations. However, urban facilitation may also lead to the introduction of alleles that are poorly suited to urban environments (Lambert & Donihue, 2020; Miles et al., 2019). Given these competing hypotheses and the need to test for convergent nonadaptive evolutionary processes, researchers must examine and compare the plethora of different taxa living in various urban environments.

Amphibians are susceptible to subtle environmental changes and in previous studies have exhibited some of the strongest responses in gene flow and genetic drift in response to urbanization (Lourenço et al., 2017; Noël et al., 2007). In this special feature, two studies examined the nonadaptive evolutionary responses of amphibian populations to urbanization using genomic data. In the first study, Fusco et al. (2020) examined Northern two‐lined salamanders (*Eurycea bislineata*) which are native to the New York City metropolitan area and live in urban, suburban, and rural stream environments. Fusco et al. (2020) found that populations experience reduced gene flow in urban areas, and this reduction was correlated with buildings, roadways, and residential housing. Yet, despite this disturbance and reduced gene flow due to urban development, all populations maintained similar levels of genetic diversity. This study suggests that even low amounts of anthropogenic disturbance can impact gene flow (Fusco et al., 2020). Interestingly, this pattern of reduced gene flow but not reduced genetic diversity was also identified in populations of the Eastern golden frog (*Pelophylax plancyi*) in Shanghai, China (Wang, 2020). Shanghai has historically been a predominantly agricultural region, but in the last 40 years it has experienced rapid and extensive urbanization. Demographic modeling showed that declines in effective population size (Ne) were prominent and often preceded urbanization. Therefore, even when populations show evidence of reduced effective population size, this can be due to scenarios other than urban development. Despite this disturbance, moderate levels of gene flow occur across peripheral rural populations and genetic diversity is maintained across populations, but is negatively impacted with increasing urbanization (Wang, 2020).

Whereas amphibians are highly susceptible to habitat fragmentation, flying animals may be able to avoid many of the barriers that terrestrial animals face. Three studies in this special issue address the issue of the relationship between dispersal ability and gene flow. Ballare and Jha (2020) found that urban and agricultural areas do not restrict gene flow in carpenter bees at a regional scale. However, there is evidence of fine‐scale population structure as individuals in close proximity are more related to one another than by chance alone, an effect that is likely a consequence of high philopatry. Similarly, Carlen and Munshi‐South (2020) found that feral pigeons (*Columba livia*) form an almost continuous population in the heavily urban megacity of the Northeastern United States, which ranges from Boston, MA, to Washington, DC. Within this greater population, pigeons from Boston and Providence form a distinct genetic cluster and pigeons from more southern urban environments form a second genetic cluster. Their research indicates that despite heterogeneous patterns of urbanization across the Northeastern United States, the region acts almost as a single urban habitat for this highly mobile human commensal. Lastly, using genomic data from two synanthropic mammals, mice (*Peromyscus leucopus*) and bats (*Eptesicus fuscus*), Richardson et al. (2020) examined how differences in terrestrial versus aerial dispersal contributes to the effects of urbanization on evolutionary processes like gene flow. Mice, which have lower dispersal ability, had generally higher overall genetic differentiation than bats, suggesting that dispersal ability strongly and positively influences urban gene flow. Additionally, mice tend to remain near their natal sites and thus inbreeding is considerably higher within mouse populations.

### 2.4 Human–wildlife interactions

Human–wildlife interactions are an inherent part of urban areas, and these interactions have the potential to shape the evolutionary trajectories of urban wildlife. As humans concentrate in urban environments, some wildlife becomes locally extinct, whereas others find refuge in urban parks, backyards, and other green spaces. Additionally, some organisms known as human commensals (e.g., brown rats, house mice, cockroaches) accompany humans as they move around the globe (Bonhomme & Searle, 2012; Puckett et al., 2016; Vargo et al., 2014), not only surviving but dependent on human‐dominated landscapes. Together, these processes lead to a distinct mix of native and non‐native organisms in every urban environment.

Socio‐political policies and traditions also contribute to the extent of urban–wildlife interactions and its evolutionary impacts. Institutionalized racial segregation in cities (e.g., redlining, Schell, Dyson, et al., 2020), along with the frequency of garbage pick‐up and pest management, all play an important role in human–wildlife interactions. These practices are deeply influenced by local politics and attitudes toward wildlife. Expanding on this, Schell, Stanton, et al. (2020) provide insight into human–wildlife interactions and the subsequent evolutionary consequences. The expansion of urbanization inevitably leads to human–wildlife interactions that may have negative effects on both people (e.g., property damage, zoonotic disease transmission) and wildlife (e.g., automobile collisions, attacks from domestic animals). Schell, Stanton, et al. (2020) point out that wildlife managers need to consider the adaptive, nonadaptive, and phenotypically plastic responses when deciding whether to remove, relocate, or deter urban wildlife. This cross‐disciplinary perspective thoughtfully integrates the fields of urban evolution and wildlife management with socio‐political policies to explore how ecological and social processes drive evolution in urban environments.

Byers et al. (2020) took a deeper dive into a single human commensal, the Norway rat (*Rattus norvegicus*), which are found in urban environments around the world. Since human–wildlife interactions can lead to pathogen transmission, and rats in particular have infamously vectored devastating human diseases (e.g., *Yersinia pestis*), it is important to understand the eco‐evolutionary dynamics of the pathogen and host relationship. Specifically, Byers et al. (2020) sought to understand how relatedness of Norway rats and their movement across a single neighborhood in Vancouver, Canada, influence the distribution of the pathogens they carry. They found that pathogen infection was not predicted by disease status of relatives. Although rats are continuously distributed across the urban environment, rat family groups cluster within city blocks. However, this spatial distribution of rats in the urban environment does not correlate with patterns of pathogen prevalence, indicating that a specific rat genotype does not confer protection against pathogen infection.

### 2.5 Emerging themes

The majority of urban evolutionary research to date has focused on terrestrial ecosystems. Yet, our waterways are impacted directly and indirectly by anthropogenic activity and urbanization. Highlighting this emerging and understudied area of urban evolutionary study, Alter et al. (2020) present a synthesis of the myriad ways urbanization influences marine organisms from a broad swath of taxa including macroalgae, invertebrates, and fishes. The marine environment is impacted by urbanization in similar or analogous ways as terrestrial environments. Marine organisms grapple with pollution, temperature increases, anthropogenic structures, and changes to background sound and light environments. These habitat modifications have consequences for the adaptive and nonadaptive evolution of marine organisms, including shaping genetic structure across seascapes. Marine organisms also face urban challenges unique to the aquatic environment, such as oxidative stress, and temporal and spatial scales of evolutionary responses may differ starkly from terrestrial organisms. Alter et al. (2020) raise several emerging themes in urban marine research, echoing themes in terrestrial research—impacts of pollutants, convergence in trait shifts, spatial heterogeneity, the implications of genetic structure, and provide a conceptual framework for guiding future studies on urban evolution in marine systems.

Another underutilized and rich resource for urban evolutionary research can be found in natural history collections. Shultz et al. (2020) propose that natural history collections are critical for contemporary and future urban evolution studies. These collections allow researchers to directly compare organisms over time to understand phenotypic and genotypic consequences of urbanization. In their review, Shultz et al. (2020) discuss how museum collections can be leveraged and how they have been used to study urban evolution to date. The authors conclude that museum collections are infrequently used despite the great potential for the study of museum specimens to drive the urban evolution field in new and exciting directions. Nevertheless, the use of museum specimens in urban evolutionary research is hindered by deposition and archiving patterns, leading the authors to make recommendations for best practices moving forward so that future researchers can best use this important window into the past.

Finally, in the invited capstone perspective paper, Des Roches et al. (2020), a team of researchers from the Urban Eco‐Evo research coordination network (RCN), contributed a forward‐looking perspective on challenges and opportunities in the future of urban evolutionary biology. The research group focused on the integration of ecological and evolutionary dynamics in urban research and emphasized the need to incorporate human social patterns and processes as drivers of ecological and evolutionary change in plants and animals, including potential feedbacks of eco‐evolutionary dynamics onto human populations. The capstone highlights the inherent interconnectedness of human activities and behavior with ecological and evolutionary processes in urban environments, and proposes a “socio‐eco‐evolutionary” framework for studying urban ecosystems.

## 3 CONCLUSION

While the 16 studies in this issue have pushed the field in new directions, they also highlight gaps that should be priorities for the future. One of these gaps is the need for widening the geographic focus. We made an open call for submissions, yet most of the studies included in this special issue are from North America and Europe, with just one study from Asia. With extreme urbanization occurring on all inhabited continents, we notice a severe lack of geographic diversity, not only in this special issue but in the literature on urban evolutionary biology more generally. We call on funding organizations and journals to prioritize urban evolution work being conducted in Africa, Asia, Australia, and South America. We urge North American and European collaborators who wish to conduct urban evolution research outside of their local region to work with local researchers who can call upon their vast knowledge of how urban socio‐eco‐evolutionary processes have shaped the landscape of urban areas. Additionally, we encourage urban evolution research networks to incorporate researchers already conducting this work locally in Africa, Asia, Australia, and South America into ongoing discussions, reviews, and future work. In addition to sampling broader geographic regions, museum collections are also needed to increase the breadth of organisms and ecosystems studied. This gap was highlighted by Shultz et al. (2020) and demonstrates the need for museum collections across all six human‐inhabited continents to actively collect specimens from urban and nonurban areas to document current organisms and provide a wealth of samples for future scientists to ask questions about. Addressing these gaps will greatly advance our understanding of urban evolutionary biology.

The research presented here also emphasizes four emerging themes that are important avenues for the future of urban evolutionary research. (1) Ectotherms have long been models for understanding thermal physiology (Angilletta, 2009), but invertebrates are now emerging as models for urban physiological evolution in particular. By building on the growing body of literature on urban thermal physiology in invertebrates to include other organisms (e.g., plants, microbes), future research will shed light on the repeatability and universality of adaptive responses to urban heat island as well as the mechanistic underpinnings of phenotypic plasticity versus genetic adaptation. (2) Several of the studies in this special issue expand our understanding of adaptive trait variation by linking it to organismal and ecosystem function. Going forward, we suggest researchers should continue to tie trait–environment variation to fitness, performance, and ecosystem level effects in an urban context, but studies should also consider urban heterogeneity (both within and between cities) and the many different drivers of adaptive change rather than rely on the same simplified metrics. (3) Genetic studies are still trying to uncover how urban fragmentation and facilitation models of gene flow shape evolutionary processes. Future research should continue to examine how different spatial scales, life history (e.g., commensal versus. noncommensal), and dispersal ability shape the spatial genetic patterns of urban organisms. (4) It is now undeniably clear that we cannot examine urban evolutionary patterns and processes without considering the inextricable human element. This means studying wildlife conflict and disease, but also integrating socioeconomic dynamics more broadly into studies of urban evolution. Looking to the future, researchers should strive to incorporate the ideas presented in Des Roches et al. (2020) by considering the ways in which their study organisms interact with humans and how variation in human social structures influence urban eco‐evolutionary dynamics. In addition, we would specifically like to highlight human–wildlife interactions, because ultimately, cities are designed for human needs but are inherently interconnected to local wildlife. Building on these themes will help advance our understanding of the multifarious socio‐eco‐evolutionary processes that drive human–wildlife interactions to better serve both humans and urban organisms.

## Data Availability

This manuscript does not have associated data.
